# Discriminant analysis and machine learning approach for evaluating and improving the performance of immunohistochemical algorithms for COO classification of DLBCL

**DOI:** 10.1186/s12967-019-1951-y

**Published:** 2019-06-11

**Authors:** Yocanxóchitl Perfecto-Avalos, Alejandro Garcia-Gonzalez, Ana Hernandez-Reynoso, Gildardo Sánchez-Ante, Carlos Ortiz-Hidalgo, Sean-Patrick Scott, Rita Q. Fuentes-Aguilar, Ricardo Diaz-Dominguez, Grettel León-Martínez, Verónica Velasco-Vales, Mara A. Cárdenas-Escudero, José A. Hernández-Hernández, Arturo Santos, José R. Borbolla-Escoboza, Luis Villela

**Affiliations:** 1 Escuela de Ingeniería y Ciencias, Tecnologico de Monterrey, Ave. Eugenio Garza Sada 2501, 64849 Monterrey, NL Mexico; 2 Escuela de Medicina y Ciencias de la Salud, Tecnologico de Monterrey, Ave. Morones Prieto 3000, 64710 Monterrey, NL Mexico; 3 Department of Bioengineering, University of Texas at Dallas, Richardson, TX USA; 4 Universidad Politécnica de Yucatán, Tablaje Catastral 4448, Carretera Mérida-Tetiz. Km.4.5., 97357 Ucú, Yucatán Mexico; 5 Department of Pathology, Hospital y Fundación Medica Sur, 14050 Mexico City, Mexico; 6 Department of Pathology, Instituto de Seguridad y Servicios Sociales de los Trabajadores del Estado (ISSSTE), Hospital General Tacuba, Lago Ontario 36, Tacuba, 11410 Mexico City, Mexico; 7 Department of Pathology, Hospital Angeles Lomas, Col. Valle de las Palmas, Hacienda de las Palmas, 52763 Huixquilucan, Mexico; 8 Querétaro, Mexico; 9 Novartis, East Hanover, NJ USA; 10 Centro Médico “Dr. Ignacio Chávez”. ISSSTESON, 83000 Hermosillo, SON Mexico; 11 Universidad del Valle de México, 83165 Hermosillo, SON Mexico

**Keywords:** DLBCL, COO identification, IHC algorithm, Machine learning, Linear discriminant analysis

## Abstract

**Background:**

Diffuse large B-cell lymphoma (DLBCL) is classified into germinal center-like (GCB) and non-germinal center-like (non-GCB) cell-of-origin groups, entities driven by different oncogenic pathways with different clinical outcomes. DLBCL classification by immunohistochemistry (IHC)-based decision tree algorithms is a simpler reported technique than gene expression profiling (GEP). There is a significant discrepancy between IHC-decision tree algorithms when they are compared to GEP.

**Methods:**

To address these inconsistencies, we applied the machine learning approach considering the same combinations of antibodies as in IHC-decision tree algorithms. Immunohistochemistry data from a public DLBCL database was used to perform comparisons among IHC-decision tree algorithms, and the machine learning structures based on Bayesian, Bayesian simple, Naïve Bayesian, artificial neural networks, and support vector machine to show the best diagnostic model. We implemented the linear discriminant analysis over the complete database, detecting a higher influence of BCL6 antibody for GCB classification and MUM1 for non-GCB classification.

**Results:**

The classifier with the highest metrics was the four antibody-based Perfecto–Villela (PV) algorithm with 0.94 accuracy, 0.93 specificity, and 0.95 sensitivity, with a perfect agreement with GEP (κ = 0.88, *P* < 0.001). After training, a sample of 49 Mexican-mestizo DLBCL patient data was classified by COO for the first time in a testing trial.

**Conclusions:**

Harnessing all the available immunohistochemical data without reliance on the order of examination or cut-off value, we conclude that our PV machine learning algorithm outperforms Hans and other IHC-decision tree algorithms currently in use and represents an affordable and time-saving alternative for DLBCL cell-of-origin identification.

**Electronic supplementary material:**

The online version of this article (10.1186/s12967-019-1951-y) contains supplementary material, which is available to authorized users.

## Background

Diffuse large B-cell lymphoma (DLBCL), also known as aggressive lymphoma, is the most common type of Non-Hodgkin lymphoma, and it can be classified into germinal center (GCB), activated B-cell (ABC), and mediastinal large B-cell lymphoma cell-of-origin (COO), the latest frequently called as unclassified (UC). The importance of these entities lies in the different driven intracellular oncogenic signaling pathways that lead to a distinct clinical outcome [1, 2]. Drugs currently under clinical trials targeting the DLBCL COO groups are being tested to provide more specific therapeutic strategies, particularly for non-germinal center-like patients who present the worse outcome [3].

The development of techniques to assess the gene expression profiling (GEP) has allowed DLBCL COO classification. GEP is now widely considered to be the “gold standard,” but its generalized clinical application remains limited due to technical, financial and regulatory obstacles [4]. The requirement of fresh tissue, large initial capital costs, an ongoing necessity for skilled labor and consumables are prohibitive to many health institutions and diagnostic laboratories [5]. Because of this current impracticality of performing GEP assays, immunohistochemistry (IHC)-decision tree algorithms for DLBCL classification have been proposed to do COO identification economical and less time-consuming. Furthermore, GCB and non-GCB COO groups have been included as representative DLBCL subtypes in the 2016 revision of the World Health Organization classification of lymphoid neoplasms, where the use of IHC is suggested [3]. Thus, there is now an impending need for accurate, robust and affordable methods to identify COO groups [6].

Hans et al. [7] established the first non-automatic IHC-decision tree algorithm for DLBCL COO classification. This algorithm was constructed by a dichotomous classifier, with activated B-cell and mediastinal large B-cell lymphoma simplified as non-germinal center-like (non-GCB) molecular subgroup. Hans algorithm uses CD10, BCL6, and MUM1 expression to classify DLBCL into GCB (CD10 and/or BCL6 positive, MUM1 negative) or non-GCB subtype with the reverse pattern of staining. Reproducibility of this algorithm leads to research groups to propose alternate IHC strategies, such as Colomo [8], Nyman [9], Choi [10], Hans modified (Hans*) [11], modified Choi (Choi*) [11] and Visco–Young [12] with three (VY3) and four (VY4) antibodies, all reported to be predictive of outcomes. All these algorithms are based on a classical decision tree structure (Additional file 1: Figure S1), and use two to five antibodies, such as CD10, BCL6, FOXP1, GCET1, and MUM1. Additionally, IHC algorithms using different antibodies have been reported [11, 13, 14].

Immunohistochemistry has been compared to GEP with regards to classification and prediction of clinical outcomes. Some studies report that IHC-decision tree algorithms correlate well with GEP in predicting prognosis [7, 10, 11], but others find a lack of prognostic utility in IHC-based COO assignment [15]. The conflicting concordance between IHC and GEP results may partly be due to the heterogeneity of studied samples (extranodal vs nodal DLBCL) and populations (treatment, patient age). Moreover, technical differences related to IHC analysis such as antigen retrieval, non-standardized scoring criteria for IHC, different primary antibodies employed in diagnostic laboratories, and batch-to-batch antibody variations may also be significant [5, 16]. Others have tried to improved IHC algorithms with biomarker studies, but this implies an elevated cost in diagnostics [17–19]. Thereby, we decided to carry out a statistical description by discriminant analysis and to explore machine learning approach to increase IHC-based classification and GEP concordance.

### Linear discriminant analysis

Linear discriminant analysis (LDA) is frequently applied to obtain a description of separability in a dataset and overall to evaluate the influence of each feature (predictor) over such separation of groups. LDA is a common analysis presented in cancer’s basic and clinical research to evaluate the data clustering [20, 21] including lymphoma studies [22]. In this study, LDA was applied to statistically describe the influence of the combination of the antibodies over the classification of GCB and non-GCB cases.

### Classification by machine learning

Machine learning algorithms have been applied for neoplastic molecular subtype classification considering as input information derived from microarrays [23], and q-PCR [24].

In this study, we applied the machine learning approach, considering as features the same combination of antibodies as in each published IHC-decision tree algorithms, following Bayesian classifier (B), Bayesian simple classifier (BS), Naïve Bayesian classifier (BN), artificial neural networks (ANN), and support vector machines (SVM) structures. Bayesian classifier considers the minimal probability of misclassification. Bayesian simple classifier involves a conditional probability model which uses independent variables, whereas Naïve Bayesian classifier considers that each feature contributes independently to the probability of the class from any other feature. ANN combines training, learning and nonlinear functions to minimize the classification error. SVM builds a model that separate each category by a gap where new sets can be predicted to belong to a category depending on which side of the gap they fall on [25]. As will be discussed, the use of automatic classification methods based on machine learning is an option to analyze the concordance and increase the accuracy between IHC and GEP. Here, we performed the first quantitative comparison between the most common nonautomatic IHC-decision tree algorithms against machine learning algorithms to derive the best diagnostic model.

## Methods

### IHC-decision trees and linear discriminant analysis of the public DLBCL database

Visco et al. [12] generated a database (from now called Visco–Young database) containing GEP, immunohistochemistry staining data (corresponding to CD10, BCL6, FOXP1, GCET1, and MUM1 antibodies), and clinical information of 475 de novo DLBCL patients who were treated with rituximab-CHOP chemotherapy (available at https://www.nature.com/articles/leu201283#supplementary-information).

Immunohistochemistry staining data of Visco–Young database was used to classify the DLBCL cases according to the eight reported IHC-decision tree algorithms, (decision trees with antibodies combination and cut-off values are detailed on Additional file 1: Figure S1). Classes were identified as GCB or non-GCB cell-of-origin groups, the later comprised by both activated B-cell (ABC) and unclassified (UC) cases. From here on, predicted classes are the results obtained for any classifier, and true classes consisted of the genetic expression profiling. To differentiate between GCB and non-GCB class, we defined the true positive (TP) result when a subject belonging to the true GCB class is identified as GCB and true negative (TN) result when a subject belonging to the true non-GCB class is identified as non-GCB.

We implemented LDA to describe the influence of each antibody over the linear separability of the database into the GCB and non-GCB classes. Considering numerical tags for each antibody: 1 = CD10, 2 = BCL6, 3 = FOXP1, 4 = GCTE1, and 5 = MUM1. We analyzed 25 possible combinations of the set of five antibodies as predictor sets; it includes the cases of such used by each one of the eight IHC-decision tree algorithms (Additional file 1: Table S1). For example, Nyman’s algorithm includes FOXP1 and MUM1, it corresponds to the predictor set combination (3,5). Colomo and Hans use the same antibody combination (1,2,5). IHC algorithms include different cut-offs but this condition is indistinct for LDA, and combinations such as (1,2) not used for any of the IHC-decision trees but assessed in the LDA analysis.

The corresponding linear discriminant function (LDF) for each combination and each class is obtained with the weight associated to each antibody. IHC-decision trees were executed in scripts for Matlab (R2018a, MathWorks, USA) and LDA analysis were performed in Minitab (v18.1, Minitab, Inc., State College, PA, USA).

### IHC-decision trees and LDA classification, comparative performance analysis

The performance of IHC-decision trees and LDA classification algorithms were evaluated by computing metrics such as accuracy (Acc), specificity (Spec), sensitivity (Sens), positive predictive value (PPV), negative predictive value (NPV), likelihood ratio for positive test results (LR+), likelihood ratio for negative test result (LR−) [26].

### Classification of DLBCL in COO molecular subgroups by automatic classifiers

For the classification stage by machine learning algorithms, the Visco–Young database was split into training, testing, and validation data subsets (75%, 20% and 5% respectively). To preserve the same proportion of ranked patients and avoid the overfitting, the so-called *k*-fold cross-validation technique was applied. Testing and validation data subsets (VY subset) were merged to assess classification performance.

Each machine learning structure, B, BS, BN, ANN, and SVM was implemented considering as feature inputs the same antibodies combinations used by the IHC-decision trees. We obtained 35 algorithms for automatic classification, considering that Colomo and Hans’s algorithms use the same combination of antibodies. As was defined, classes were identified as GCB or non-GCB COO groups. To avoid overfitting, we used large training set (75% of cases as mentioned above); we implemented *k*-fold cross-validation to assure that classification results were independent of the training, testing, and validations data subsets; and during training, a level of 95% maximum accuracy was set. The stopping criterion during the training stage for all classification models was an error less than 1 × 10^−3^ or 100 training epochs, whichever was satisfied first.

Bayesian classifiers were executed in Weka [27]. Artificial neural networks were implemented using Matlab Neural Network Pattern Recognition Toolbox. In the hidden layer, five neurons were used, and the cross-validation method selected was Entropy Reduction. Support vector machines were implemented using the Sci-Kit Learn Python Library [28], with a Kernel Transformation using the Radial Base Function, and using stratified *k*-fold grid search cross-validation. Classification of data can be done upon request, and a mobile application is in the making.

### IHC-decision trees and machine learning classifiers, comparative performance analysis

The same metrics used for comparison between IHC and LDA were calculated to compare the IHC-decision trees and trained machine learning algorithms, for both approaches the VY subset is considered for classification comparison. Additionally, to statistically asses the concordance between IHC and machine learning algorithms with the GEP true class, all outputs were analyzed by Cohen’s kappa (κ) for agreement analysis and Pearson’s Chi-squared test in Matlab. Kappa result was interpreted as follows: values ≤ 0 as indicating no agreement and 0.01–0.20 as poor, 0.21–0.40 as fair, 0.41– 0.60 as moderate, 0.61–0.80 as good, and 0.81–1.00 as very good agreement. Per example, a κ = 0.01 comparing GEP to a model indicated no agreement, that is, a GCB patient identified by GEP was misclassified as non-GCB by an algorithm. Conversely, a κ = 0.85 indicated very good agreement, that is, the GCB patient identified by GEP was correctly classified as GCB by an algorithm.

### Validation in a clinical sample set

To test the efficacy of COO molecular group classification, we use an independent series of cases. Archived formalin-fixed, paraffin-embedded (FFPE) tissues from 60 DLBCL Mexican-mestizo patients were collected from January 2009 to March 2011, after approval by the Institutional Review Board of Escuela de Medicina y Ciencias de la Salud from Tecnologico de Monterrey. Retrieved blocks dated from 1999 to 2010. Clinical data were incomplete for 11 cases; therefore, they were withdrawn from the analysis.

FFPE sections from clinical sample set patients were stained with standard hematoxylin–eosin (H&E) staining method. Each H&E slide was reviewed, and morphologically representative, non-necrotic tumor areas were used for tissue array (TA) assembly.

Immunohistochemical analysis was performed on 3 µm thickness TA sections using a streptavidin–biotin complex technique. The antibodies utilized were FOXP1 (abcam, clone JC2 at 1:300 dilution), GCET1 (abcam, clone RAM341 at 1:100 dilution), CD10 (Santa Cruz Biotechnology, clone 56C6 at 1:10 dilution), BCL6 (Santa Cruz Biotechnology, clone 0.N.26 at 1:5 dilution), and MUM1 (Santa Cruz Biotechnology, monoclonal, at 1:50 dilution). Each array slide was mounted with tonsil as positive control tissue. A comparison with the antibodies and dilutions used by Visco et al. is shown in Additional file 1: Table S2.

Three pathologists reviewed the slides independently. Difficult cases were examined and resolved by joint review on a multiheaded microscope. Such an event occurred in less than 5% of the cases. Immunoreactivity was scored as the percentage of positive tumor cells over total tumor cells. The data analysis included only the cases with both complete IHC scores readings and clinical information.

### Survival analysis

For VY subset and clinical sample set, survival of GCB and non-GCB COO groups identified by all algorithms were compared using Kaplan–Meier curves, and the significance was calculated using log-rank test. For all the analysis, a *p*-value < 0.05 was considered to be statistically significant.

## Results

### Public database analysis

Visco–Young database comprised 231 GCB, 200 activated B-cell (ABC), and 44 unclassified (UC) cases identified by genetic profiling, resulting in 231 (48.6%) GCB and 244 (51.4%) non-GCB cases. In Visco–Young database, 71% of the cases occurred in patients from North America, 6% from Asia, and the remainder were from Western Europe (Italy, Spain, Switzerland, Denmark, and The Netherlands). The median age was 62 years (61% > 60 years, and male patients ~ 57%). Patients were in advanced Ann Arbor stage (53%), with not favorable performance status (20% with ECOG ≥ 2), high lactate dehydrogenase values in serum (65%), with extranodal involvement (23% with ≥ 2), and with B-symptoms manifestations (32%). All these characteristics accounted for 14% patients having a high intermediate to high IPI risk. Treatment consisted of R-CHOP (91%) or R-CHOP-like regimens (9%; epirubicin or mitoxantrone based combination chemotherapy). Patients that achieved complete remission on initial chemotherapy treatment were 76%.

For automatic classifiers, a total of 354 cases were used during training, and the remaining 121 (VY subset) were used for testing and validation steps. This VY subset included 51.2% GCB and 48.8% non-GCB by GEP, a similar proportion to the training set, and was used to compare classification metrics.

### Clinical sample set characteristics

Our clinical sample set comprised of Mexican-mestizo patients (see Additional file 1: Table S3) is similar to Visco–Young database. Main treatment was anthracycline-based (36.7%), R-anthracycline-based (42.9%), and 20.4% as other chemotherapeutic regimens. It is important to mention that around 57% of the patients did not receive Rituximab since this treatment is not available in the entire public Mexican health system. In regards with response, almost half of the patients achieved complete remission (49.0%) on initial chemotherapy treatment, and 12.2% of them relapsed during follow-up. Median follow-up time was 30.3 months, and 63.3% of the patients had a follow-up above 9 months.

Even though the ethnic background of these sets is different, clinical characteristics were comparable. Regarding antibodies used, we used the same clones than Visco et al. for FOXP1, CD10, and GCTE1. Additionally, although most patients were not treated with Rituximab as in Visco et al., early reports of DLBCL COO classification used samples from patients treated with mainly anthracycline-based chemotherapy [29–31].

### IHC-decision trees and classification by LDA, performance analysis

The metrics obtained for each IHC-decision tree considering the complete classification of the cases in the Visco–Young database are presented in Table 1. In the same table we show the equivalent classification after the LDA for the same antibody combination, according to the derived linear discriminant function (LDF). We have also included the most relevant combination that has not been evaluated by any IHC-decision tree algorithms. The complete classification metrics for the rest of possible combinations by LDA and their respective set of linear coefficients can be consulted in Additional file 1: Tables S4, S5, respectively.

**Table 1:** Performance metrics of classification of IHC-decision tree algorithms and LDA

	Algorithm	Antibody combination	Acc	Sens	Spec	PPV	NPV	LR+	LR−
IHC-decision trees	Nyman	3,5	0.72	0.52	0.91	0.84	0.67	5.56	0.53
	Colomo	1,2,5	0.78	0.71	0.84	0.81	0.75	4.56	0.34
	Hans	1,2,5	0.85	0.91	0.78	0.80	0.91	4.21	0.11
	Hans*	1,5	0.82	0.94	0.70	0.75	0.92	3.14	0.09
	Choi	1,2,3,4,5	0.88	0.94	0.84	0.84	0.93	5.70	0.08
	Choi*	1,3,4,5	0.79	0.74	0.83	0.80	0.77	4.30	0.31
	VY3	1,2,3	0.88	0.92	0.84	0.85	0.92	5.92	0.09
	VY4	1,2,3,4	0.88	0.93	0.84	0.85	0.92	5.80	0.09
Linear discriminant analysis	As in Hans*	1,5	0.84	0.77	0.91	0.89	0.81	8.59	0.25
	As in Nyman	3,5	0.77	0.81	0.74	0.75	0.81	3.10	0.25
	As in VY3	1,2,3	0.89	0.87	0.91	0.90	0.88	9.19	0.15
	As in Hans/Colomo	1,2,5	0.87	0.86	0.88	0.87	0.87	7.25	0.16
	–	1,4,5	0.87	0.81	0.92	0.90	0.84	9.93	0.20
	As in VY4	1,2,3,4	0.87	0.84	0.90	0.89	0.86	8.24	0.17
	As in Choi*	1,3,4,5	0.88	0.86	0.91	0.90	0.87	9.09	0.16
	As in Choi	1,2,3,4,5	0.89	0.87	0.91	0.90	0.88	9.23	0.14

The upper section corresponds to the performance of the IHC-decision tree algorithms. Lower section corresponds to equivalent combinations of antibodies, but with LDA classification, this includes the rest of combinations not reported by IHC-decision tree algorithms. Choi, VY3, and VY4 algorithms reached the most considerable accuracy, representing the most balanced options of sensibility and specificity, with similar performance metrics

Numeric tags 1 = CD10, 2 = BCL6, 3 = FOXP1, 4 = GCTE1, and 5 = MUM1

Acc: accuracy; Sens: sensitivity; Spec: specificity; PPV: positive predictive value; NPV: negative predictive values; LR+: likelihood ratio for positive test results; LR−: likelihood ratio for negative test result

### IHC-decision tree algorithms performance

Three algorithms Choi, VY3, and VY4, reached the most considerable accuracy (Acc 0.88), representing the most balanced options of sensibility and specificity, with similar performance metrics (Table 1). These algorithms presented a better performance for the classification of GCB subjects (Sens 0.94 Choi, 0.92 VY3, 0.93 VY4) compared with non-GCB classification (Spec 0.84); however, regarding to the probability that a subject classified as GCB was correctly classified, the outcome is fair (PPV 0.84 Choi, 0.85 VY3, 0.85 VY4), but certainly better for non-GCB (NPV 0.93 Choi, 0.92 VY3, 0.92 VY4). Similar results were obtained for likelihood ratios, moderate for positive test results (LR+ 5.70 Choi, 5.92 VY3, 5.80 VY4) and excellent for negative test results (LR− 0.08 Choi, 0.09 VY3, 0.09 VY4). It is important to remark that VY3 considers only three antibodies (1,2,3), such combination is included in Choi and VY4 with a different cut-off in the decision tree branches. Nyman reached the best specificity (Spec 0.91); however, the probability that a subject classified as non-GCB was correctly classified is low (NPV 0.67), its LR− is also reduced (LR− 0.53). Hans algorithm, usually considered as a reference, had lower performance compared with VY3, VY4, and Choi. The worst evaluated were Colomo and Choi*, and the excellent sensitivity of Hans* (Sens 0.94) it is not enough to match the performance of Choi, VY3, and VY4.

### Linear discriminant analysis performance

Antibody combinations (1,2,3) and (1,2,3,4,5) reached the more considerable accuracy (Acc 0.89), these combinations are the same used by VY3 and Choi respectively. For these linear discriminants the GCB classification gives a fair sensibility (Sens 0.87), which is lower than sensitivity for the equivalent IHC-decision trees; but, in terms of probability their PPV is better (PPV 0.90), and the likelihood ratio for LR+ (LR+ 9.19 (1,2,3), 9.23 (1,2,3,4,5)) overcome the performance of VY3 and Choi. This can be appreciated on Additional file 1: Figure S2, where the separation of COO groups increases using LDA in comparison to IHC-decision trees.

In accordance to results in Table 2, for combination (1,2,3), the higher coefficients of the LDF for GCB cases relays on antibody BCL6 = 6.99 and is followed by CD10 = 4.86, with less influence of FOXP1 = 0.64. A similar situation is for combination (1,2,3,4,5), its coefficient for BCL6 = 6.31 and is followed by CD10 = 4.59; less impact is obtained from the rest of antibodies (CGET1 = 3.28, MUM1 = 1.02 and FOXP1 = 0.71). As can be appreciated in Table 2, the inclusion of BCL6 and CD10 in the LDF is strongly related to the classification of GCB cases, for any of the combinations in which they are included, their coefficients are the largest; keeping the coefficient relation: BCL6 larger than CD10 coefficient if they occur in the same combination. Antibody combinations (1,2,3) and (1,2,3,4,5) have a larger specificity (Spec 0.91); however, their NPV and LR− cannot overcome their counterparts IHC-decision trees. On average sense, performance metrics are improved by LDA classification for GCB classification, both diminish the non-GCB classification metrics. From Table 2, it is possible to confirm that the inclusion of MUM1 in any algorithm is related to non-GCB detection, getting the most considerable value in the LDF.

**Table 2:** Coefficients of linear discriminant functions (LDF) derived from LDA for all possible combination of antibodies

Antibody combination		Sens	Spec	COO	Constant	Antibody				
						1	2	3	4	5
						CD10	BCL6	FOXP1	GCET1	MUM1
As in Nyman	3,5	0.81	0.74	GCB	− 0.57			2.47		1.11
				Non-GCB	− 3.29			4.38		5.06
As in Hans and	1,2,5	0.86	0.88	GCB	− 4.21	5.01	7.00			1.05
As in Colomo				Non-GCB	− 3.09	0.20	4.99			5.69
As in Hans*	1,5	0.77	0.91	GCB	− 2.29	6.53				2.11
				Non-GCB	− 2.12	1.28				6.45
As in Choi	1,2,3,4,5	0.87	0.91	GCB	− 4.80	4.59	6.31	0.71	3.28	1.02
				Non-GCB	− 3.98	− 0.41	3.80	3.75	1.24	4.73
As in Choi*	1,3,4,5	0.86	0.91	GCB	− 3.34	5.67		1.78	4.03	1.67
				Non-GCB	− 3.46	0.24		4.39	1.69	5.12
As in VY3	1,2,3	0.87	0.91	GCB	− 4.18	4.86	6.99	0.64		
				Non-GCB	− 2.90	− 0.74	4.58	4.61		
As in VY4	1,2,3,4	0.84	0.90	GCB	− 4.75	4.49	6.44	0.91	3.26	
				Non-GCB	− 2.97	− 0.86	4.40	4.70	1.12	
–	1,4,5	0.81	0.92	GCB	− 3.14	6.01			3.93	2.22
				Non-GCB	− 2.23	1.09			1.44	6.49

Columns (Antibody) show the coefficient associated with each antibody for GCB classification (Sensibility performance) and non-GCB classification (Specificity performance). The first column remarks the combinations of antibodies as were used by IHC-decision trees. BCL6 and CD10 in the LDF are strongly related to the classification of GCB cases, whereas the inclusion of MUM1 in any algorithm is related to non-GCB detection, getting the most considerable value in the LDF

Numeric Tags 1 = CD10, 2 = BCL6, 3 = FOXP1, 4 = GCTE1, and 5 = MUM1

By LDA, we observed that BCL6 coefficient weight resembles with the molecular hallmark of GCB, since BCL6 expression is mainly restricted to germinal center cells [32, 33]. Moreover, CD10 has been reported as been positive and negative for GCB patients [18, 34, 35], and appeared to have higher influence for GCB identification according to the CD10 coefficient weight we obtained. Although in normal GC B-cells MUM1 and BCL6 are mutually exclusive, in tumor cells both proteins are coexpressed [36], and we observed a more significant influence of MUM1 for non-GCB group, in agreement with it post-germinal marker feature [18].

We want to remark that there is not any combination with better metrics than those usually used by the IHC-decision trees. For that reason, machine learning approach was focused on the equivalent antibody combinations proposed by previous authors.

### Machine learning algorithms performance analysis

The metrics of the best five of 35 machine learning and 8 IHC-decision tree algorithms in VY subset are shown in Table 3. Complete results of 43 algorithms are shown in Additional file 1: Table S6.

**Table 3:** Metrics of IHC-decision tree and machine learning algorithms

	Algorithm	Antibody combination	Acc	Sens	Spec	PPV	NPV	LR+	LR−
IHC-decision tree	Nyman	3,5	0.79	0.65	0.95	0.93	0.71	12.47	0.37
	Colomo	1,2,5	0.84	0.77	0.91	0.91	0.79	8.98	0.25
	Hans	1,2,5	0.89	0.95	0.83	0.86	0.94	5.52	0.06
	Hans*	1,5	0.86	0.95	0.76	0.81	0.94	3.94	0.06
	Choi	1,2,3,4,5	0.93	1.00	0.84	0.87	1.00	6.44	0.00
	Choi*	1,3,4,5	0.83	0.79	0.86	0.86	0.79	5.73	0.24
	VY3	1,2,3	0.90	0.97	0.83	0.86	0.96	5.61	0.04
	VY4	1,2,3,4	0.90	0.97	0.83	0.86	0.96	5.61	0.04
Machine learning	PV	1,3,4,5	0.94	0.95	0.93	0.94	0.95	13.8	0.05
	ANN	1,2,3,4,5	0.94	0.95	0.93	0.94	0.95	13.8	0.05
	BS	1,2,3,4,5	0.94	0.95	0.93	0.94	0.95	13.8	0.05
	SVM	1,2,3,4,5	0.94	0.97	0.91	0.92	0.96	11.23	0.04
	SVM	1,2,3,4	0.94	0.97	0.91	0.92	0.96	11.23	0.04

Metrics correspondent to eight IHC-decision tree algorithms and the best five machine learning algorithms are shown, cases of the VY subset were classified. Numeric Tags 1= CD10, 2 = BCL6, 3 = FOXP1, 4 = GCTE1, and 5 = MUM1. IHC-decision tree algorithms could not overcome any of the remarkable metrics obtained for the best five machine learning algorithms

Acc: accuracy; Sens: sensitivity; Spec: specificity; PPV: positive predictive value; NPV: negative predictive values; LR+: likelihood ratio for positive test results; LR−: likelihood ratio for negative test result; PV: Perfecto–Villela; ANN: artificial neural networks; BS: Bayesian simple; SVM: support vector machine

**Fig. 1:**
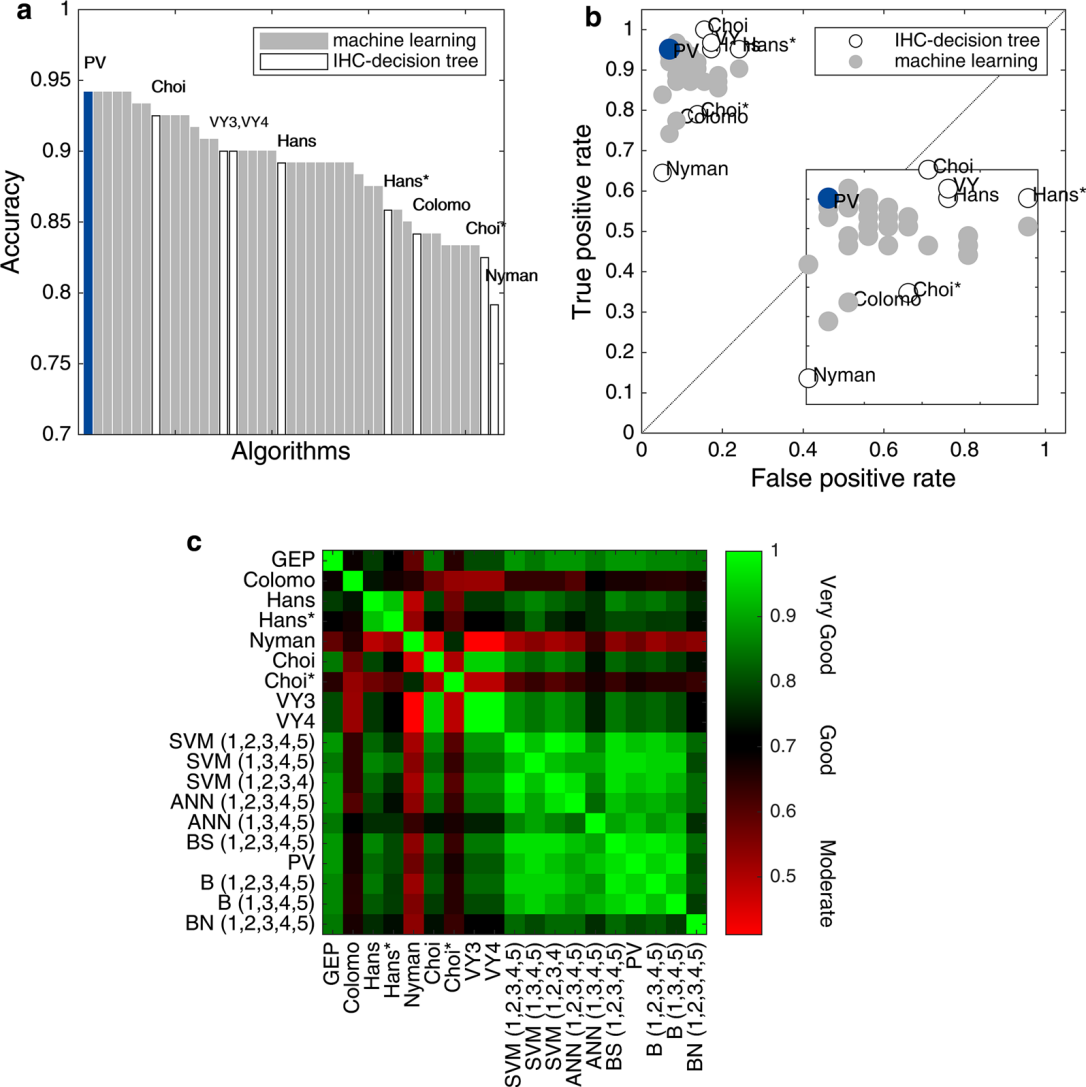
Performance and agreement comparison of IHC-decision tree and machine learning algorithms. **a** Accuracy ranking. Machine learning (gray bars) and IHC algorithms (white bars) were ordered by accuracy. PV, ANN (1,2,3,4,5), BS (1,2,3,4,5), SVM (1,2,3,4,5), and SVM (1,2,3,4) algorithms showed the highest accuracy, whereas Hans ranked in 21th place. **b** ROC space. Machine learning algorithms (gray markers), particularly PV (blue marker), ANN (1,2,3,4,5), BS (1,2,3,4,5), SVM (1,2,3,4,5), and SVM (1,2,3,4) allocated in the far left-hand side of the graph, suggesting a better performance when compared with IHC-decision tree algorithms (white markers). Nyman, Colomo and Choi* showed a more conservative performance. **c** Agreement heatmap. Scale represents moderate (0.41 ≤ κ ≤ 0.60), good (0.61 ≤ κ ≤ 0.80), to very good agreement (κ > 0.81) with red, black and green, respectively. Machine learning algorithms provided an almost perfect agreement with GEP, being ANN (1,2,3,4,5), BS (1,2,3,4,5), PV, SVM (1,2,3,4,5), and SVM (1,2,3,4) with the highest values (all with κ = 0.88, *P* < 0.001). A very good agreement within machine learning algorithms was observed (κ: 0.77–1.00). The concordance between IHC-decision tree algorithms was from moderate to good (κ: 0.41–0.79), except for Choi having a very good agreement with both VY3 and VY4 (κ = 0.95, *P* < 0.001). Numeric Tags 1 = CD10, 2 = BCL6, 3 = FOXP1, 4 = GCTE1, and 5 = MUM1. PV: Perfecto–Villela; B: Bayesian; BS: Bayesian simple; BN: Naïve Bayesian; ANN: artificial neural networks; SVM: support vector machines

Our results show Hans as a fair performance classifier and ranked 21 out of 43 algorithms tested (ranking by accuracy, Fig. 1a). Hans had a better performance for the classification of GCB subjects (Sens 0.95) compared with non-GCB classification (Spec 0.83); however, the probability that a GCB subject was correctly classified was fair (PPV 0.86), but better for non-GCB (NPV 0.94). An in terms of likelihood ratios, these were moderate for GCB (LR+ 5.52) and excellent for non-GCB (LR− 0.06). Nonetheless, Choi was the IHC-decision tree algorithm with better metrics (Acc 0.93), and ranked 8 out of 43 algorithms tested (Fig. 1a), followed by VY3 and VY4 (ranked 15 and 16, respectively). Choi presented an excellent performance for GCB classification (Sens 1.00), but with interesting metrics for non-GCB cases considering probability and likelihood ratios (NPV 1.00, LR− 0.00).

Interestingly, IHC-decision tree algorithms could not overcome any of the remarkable metrics obtained for the best five of our machine learning algorithms: Perfecto–Villela (Bayesian simple of CD10, FOXP1, GCTE1, and MUM1 combination, from now on PV algorithm), ANN (1,2,3,4,5), BS (1,2,3,4,5), SVM (1,2,3,4,5) and SVM (1,2,3,4). All of them ranked in the 1st to 5th positions out of 43 algorithms tested (Acc 0.94, Fig. 1a). PV, ANN (1,2,3,4,5), and BS (1,2,3,4,5) were excellent for both COO groups identification (Sens 0.95, Spec 0.93), with high probability of correct classification (PPV 0.94, NPV 0.95), and likelihood ratios (LR+ 13.80, LR− 0.05). Following them closely were SVM (1,2,3,4,5) and SVM (1,2,3,4) with excellent COO groups identification (Sens 0.97, Spec 0.91), with high probability of correct classification (PPV 0.92, NPV 0.96), and likelihood ratios (LR+ 11.23, LR− 0.04). It is important to notice that while some of the eight IHC-decision tree algorithms presented high PPV and NPV values, they were not predictive of COO group diagnosis as the positive likelihood ratios were low. Moreover, the best five machine learning algorithms were strongly predictive of COO group diagnosis as the positive likelihood ratios were substantially higher than 10, and their negative likelihood ratios were below 0.1, ruling out the COO group diagnosis with confidence.

It should be noted that although in LDA analysis BCL6 antibody had emerged as a relevant component in COO classification, PV algorithm does not include it. BCL6 influence in the linear discriminate is not necessary for the BS (Bayesian simple), since this structure relies on a probability function instead of a linear component. It is consistently observed in the rest of the machine learning algorithms, which are more complex structures and do not need the weight of BCL6, CD10, and MUM1 as such in simpler structures like IHC-decision tree or LDA.

Based on the ROC spaces computed for each algorithm (Fig. 1b), the best performances were obtained for machine learning algorithms, grouping mainly altogether with high true positive rate and low false positive rate. IHC-decision tree algorithms as Nyman, Colomo and Choi* showed a more conservative performance.

An agreement heatmap (Fig. 1c) of IHC-decision tree and machine learning algorithms was constructed. Hans had a good agreement (κ = 0.78, *p* < 0.001), whereas Choi had a very good agreement when compared with GEP (κ = 0.85, *p* < 0.001). Machine learning algorithms provided a very good agreement with GEP, being ANN (1,2,3,4,5), BS (1,2,3,4,5), PV, SVM (1,2,3,4,5), and SVM (1,2,3,4) with the highest values (all with κ = 0.88, *p* < 0.001). Moreover, the agreement between IHC-decision tree algorithms was from moderate to good (κ: 0.41–0.79), except for Choi having a very good agreement with VY3 and VY4 (κ = 0.95, *p* < 0.001). Conversely, a very good agreement within machine learning algorithms was observed (κ: 0.77–1.00). According to our results, PV appeared to be the best algorithm, given the use of less number of antibodies, 4 instead of 5, and good overall metrics, ensuring the investment of less time and resources.

### Survival analysis

A survival analysis with VY subset and our Mexican-mestizo clinical sample set was performed, using the eight IHC-decision tree and the 35 machine learning algorithms here proposed.

**Fig. 2:**
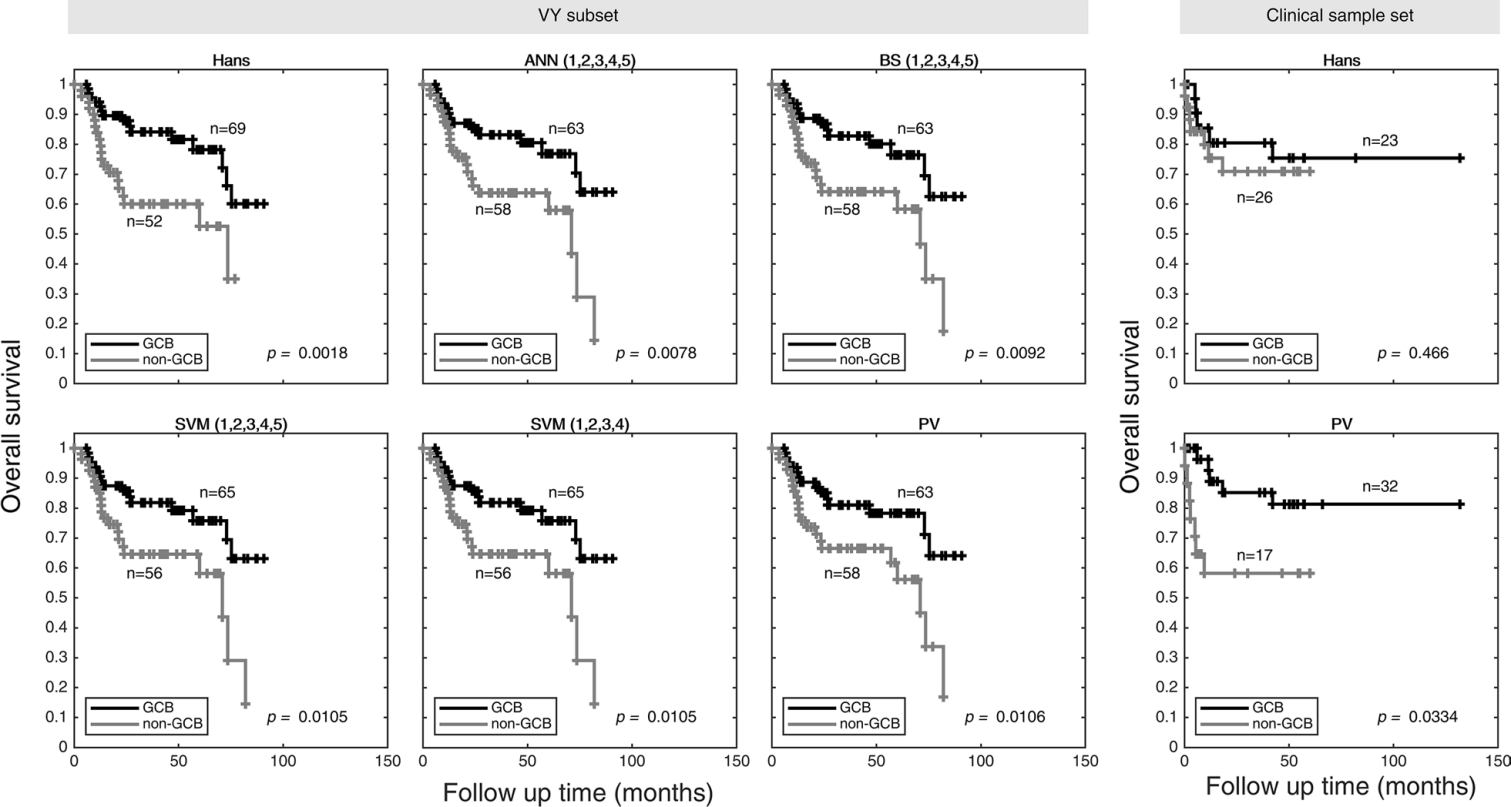
Survival analysis comparison of IHC-decision tree and machine learning algorithms. Overall survival of patients classified by Hans and the best five machine learning algorithms is shown. GCB (black) overall survival was significatively better than non-GCB (grey) cases for both VY subset and clinical sample set, except for Hans when used in clinical sample set. Numeric Tags 1 = CD10, 2 = BCL6, 3 = FOXP1, 4 = GCTE1, and 5 = MUM1. PV: Perfecto–Villela; BS: Bayesian simple; ANN: artificial neural networks; SVM: support vector machines

Kaplan–Meier curves of DLBCL COO molecular groups classified by Hans and the best five machine learning algorithms were carried out using VY subset data. As expected, GCB overall survival was significantly better than non-GCB cases (Fig. 2). Our machine learning algorithms provided a clear difference in GCB patients outcome (for PV, *p* = 0.011), along with Hans IHC-decision tree algorithm (*p* = 0.002).

In the case of our clinical Mexican-mestizo sample set, Hans IHC-decision tree algorithm classified 23 GCB patients and 26 non-GCB patients, whereas PV classification resulted in 32 GCB patients and 17 non-GCB patients (Fig. 2). The validation set of 49 cases is small but sufficient to show a statistical significant survival difference. In both scenarios, GCB patients overall survival was better (*p* = 0.466 by Hans and *p* = 0.033 by PV classification), as has been clinically observed in other populations. When we analyzed the most important clinical variables according to the COO (non-GCB vs GCB), we observed that only the high-risk group according to the NCCN-prognostic index was higher in non-GCB than GCB (23.5% vs 3.1%, respectively, *p* = 0.016). We also observed a trend of less extranodal involvement in 2 or more sites in non-GCB than GCB (29.4% vs. 40.6%, respectively, *p* = 0.07). Regarding the response after treatment (complete response vs. partial response + refractory) and the relapse, no differences were observed either. To the best of our knowledge, our study is the first analyzing and classifying a Mexican-mestizo sample set.

## Discussion

Gene expression profiling is the gold standard for DLBCL cell-of-origin classification. The presence of MYC/BCL2 translocations, overexpression of specific genes or the presence of mutations will be part of COO classification in the near future, since two large multiplatform genomic analysis have described four [37] and five [38] prominent genetic subtypes in DLBCL that will unravel the pathogenesis of the disease beyond the cell-of-origin classification. Unfortunately, GEP wide application is still prohibitive to many health institutions and diagnostic laboratories. Hans remains the most popular IHC-decision tree algorithm used for COO DLBCL classification and has a reasonable correlation with the GEP [3]. However, some issues have arisen about the proportion of misclassified cases by IHC-decision tree algorithms compared with GEP, since a higher percentage of misclassified cases in the GCB than in the non-GCB group has been observed, with proportions of up 60.0% and 38.0% for GCB and non-GCB, respectively [15, 39]. In our analysis, Hans IHC-decision tree algorithm misclassified 17.2% of GCB and 4.8% of non/GCB cases (PPV NPV). Additionally, Hans* had the worst proportions of cases that were not correctly allocated for GCB with 24.1%, whereas Nyman had the worst for non-GCB with 35.5%. Remarkably, PV had 6.9% and 4.8% of misclassified cases for GCB and non-GCB, respectively. As has been discussed by Meyer [11], this reflects that IHC-decision tree algorithms use a combination of antibodies for proteins expressed predominantly by either GCBs or non-GCB cases and examined in a specific order. Because of reliance on the order of examination, the result of an antibody early in the algorithm can make the results of antibodies used later in the algorithm irrelevant. Moreover, our LDA analysis showed higher influence over the linear discriminant function (LDF) of BCL6 and CD10 for GCB, and MUM1 for non-GCB, consistent with the hallmarks of both COOs. Furthermore, there was not an antibodies combination with better performance metrics tan such explored by IHC-decision trees; however, classification by LDF derived from LDA improved LR+ and LR− metrics. Thereby for machine learning analysis we only assessed the antibodies combination proposed by previous authors.

Coutinho et al. [15], investigated the concordance of nine IHC-decision tree algorithms for the molecular classification of a 298 sample set of DLBCL diagnostic biopsies. They used Hans, Hans*, Nyman, Choi, Choi*, VY3, VY4, Natkunam, Tally, Muris IHC-decision tree algorithms, the last three not considered in our study. They found an extremely low concordance across those nine IHC-decision tree algorithms, with only 4.1% of the tumors classified as GCB and 21.0% as non-GCB by all IHC-decision tree algorithms. Moreover, poor and fair κ values were detected in 44.4% on pairwise concordance assessment; and in only 20.0% was κ good or very good. The highest level of agreement was found between Choi, and VY3 and VY4 IHC-decision tree algorithms (κ = 0.85). GEP data for their sample set was not available, and therefore no further conclusions can be done. Considering our analysis, we found a moderate concordance across the eight IHC-decision tree algorithms here tested, with 30.0% of both GCB and non-GCB cases classified as such. Moreover, a higher concordance was observed across our five best machine learning algorithms, with 50.0% of the cases classified as GCB and 45.0% as non-GCB by the five machine learning algorithms. On pairwise concordance assessment, we observed 46.4% of IHC-decision tree algorithms with moderate κ values, 39.3% were good, and 4.0% were very good within themselves. The highest level of agreement was found between VY3 and VY4 (κ = 1.0). This can be explained since Visco [12] evaluated using the same clones. Conversely, machine learning algorithms outperformed with 8.9% good, and 91.1% very good κ values. The highest level of agreement was found between the SVM (1,2,3,4,5) and SVM (1,2,3,4) algorithms (κ = 1.0). This machine learning algorithms performance is portrayed as the abundance of green color region in the heatmap depicted in Fig. 1c, compared with the red and black regions correspondent to IHC-decision tree algorithms concordance. Nevertheless, parameters such as accuracy, sensitivity, and specificity for GEP data is a better approach to describe a classifier performance.

In the case of DLBCL, the adequate identification of GCB and non-GCB COO groups represents a major concern in personalized treatment. Nowadays, clinical guidelines for DLBCL patients includes R-CHOP as first-line treatment regardless COO group [40, 41]. It is important to point out that current clinical trials rely COO DLBCL classification on Hans IHC-decision tree algorithm, compromising final outcomes since this algorithm has less accuracy and overall performance metrics than Choi, VY3, and VY4, as we observed in our study. The lack of significant differences between GCB or non-GCB treatment outcomes using precision medicine regimens under investigation (i.e. lenalidomide, ibrutinib, and bortezomib) [42–44] is evidence of this. The machine learning approach can achieve improvement of accuracy and the rest of the performance metrics in immunohistochemistry-based COO DLBCL classification, and further study is needed. In our case, it was not possible to analyze the clinical sample set by GEP; however, the clinical protocol to test our PV algorithm is at an early stage. Translation strategy will comprise an embed process of PV algorithm in a software application, as an auxiliary tool in the diagnostic for the pathologist when GEP is not available, as well as a guide to the clinician for better treatment choices.

## Conclusions

In conclusion, our results suggest that the use of Hans should be reconsidered in favor of new classification techniques when GEP analysis is not accessible. By harnessing all of the available immunohistochemical data without reliance on the order of examination or a cut-off value, we demonstrated that even linear discriminant analysis allowed to improve the performance metrics compared to IHC-decision tree. Furthermore, automatic classification approach based on machine learning algorithms with more complex decision functions are a competitive alternative that facilitates the classification problems in a clinical setting. It is important to mention that this is the first study using a machine learning approach to improve DLBCL COO identification by IHC, and represents a promising, affordable and time-saving alternative to common IHC-based decision tree algorithms. Testing with a larger clinical sample set with GEP data will enforce the validity of this result.

## Additional file

**Additional file 1.** Additional Figures S1, S2 and Tables S1–S6.

## Data Availability

Visco et al. database used for this work is available at https://www.nature.com/articles/leu201283#supplementary-information. The Mexican-Mestizo dataset used and/or analyzed during the current study is available from the corresponding author on reasonable request.

## Abbreviations

Acc: accuracy
ABC: activated B-cell like
ANN: artificial neural networks
B: Bayesian classifier
BS: Bayesian simple classifier
COO: cell-of-origin
DLBCL: diffuse large B-cell lymphoma
ECOG: Eastern Cooperative Oncology Group
FFPE: formalin fixed paraffin embedded tissue
GCB: Germinal center B-cell like
GEP: gene expression profiling
H&E: hematoxylin–eosin
IHC: immunohistochemistry
LR−: likelihood ratio for negative test
LR+: likelihood ratio for positive test
LDA: linear discriminant analysis
LDF: linear discriminant function
BN: Naïve Bayesian classifier
NPV: negative predictive value
non-GCB: non-germinal center B-cell like
PV: Perfecto–Villela classifier
PPV: positive predictive value
Sens: sensitivity
Spec: specificity
SVM: support vector machine classifier
TA: tissue array
TN: true negative
TP: true positive
UC: unclassified
VY: Visco–Young

## References

[1] Fang C, Xu W, Li J-Y (2010). A systematic review and meta-analysis of rituximab-based immunochemotherapy for subtypes of diffuse large B cell lymphoma. Ann Hematol.

[2] Lenz G, Wright GW, Emre NCT, Kohlhammer H, Dave SS, Davis RE (2008). Molecular subtypes of diffuse large B-cell lymphoma arise by distinct genetic pathways. Proc Natl Acad Sci.

[3] Swerdlow SH, Campo E, Pileri SA, Harris NL, Stein H, Siebert R (2016). The 2016 revision of the World Health Organization classification of lymphoid neoplasms. Blood.

[4] Caimi PF, Hill BT, Hsi ED, Smith MR (2016). Clinical approach to diffuse large B cell lymphoma. Blood Rev.

[5] Gifford GK, Gill AJ, Stevenson WS (2016). Molecular subtyping of diffuse large B-cell lymphoma: update on biology, diagnosis and emerging platforms for practising pathologists. Pathology.

[6] Scott DW (2015). Cell-of-origin in diffuse large B-cell lymphoma: are the assays ready for the clinic?. Am Soc Clin Oncol Educ Book.

[7] Hans CP, Weisenburger DD, Greiner TC, Gascoyne RD, Delabie J, Ott G (2004). Confirmation of the molecular classification of diffuse large B-cell lymphoma by immunohistochemistry using a tissue microarray. Blood.

[8] Colomo L, López-Guillermo A, Perales M, Rives S, Martínez A, Bosch F (2003). Clinical impact of the differentiation profile assessed by immunophenotyping in patients with diffuse large B-cell lymphoma. Blood.

[9] Nyman H, Adde M, Karjalainen-Lindsberg M-L, Taskinen M, Berglund M, Amini R-M (2007). Prognostic impact of immunohistochemically defined germinal center phenotype in diffuse large B-cell lymphoma patients treated with immunochemotherapy. Blood.

[10] Choi WWL, Weisenburger DD, Greiner TC, Piris MA, Banham AH, Delabie J (2009). A new immunostain algorithm classifies diffuse large B-cell lymphoma into molecular subtypes with high accuracy. Clin Cancer Res.

[11] Meyer PN, Fu K, Greiner TC, Smith LM, Delabie J, Gascoyne RD (2011). Immunohistochemical methods for predicting cell of origin and survival in patients with diffuse large B-cell lymphoma treated with rituximab. J Clin Oncol.

[12] Visco C, Li Y, Xu-Monette ZY, Miranda RN, Green TM, Li Y (2012). Comprehensive gene expression profiling and immunohistochemical studies support application of immunophenotypic algorithm for molecular subtype classification in diffuse large B-cell lymphoma: a report from the International DLBCL Rituximab-CHOP Consortium Program Study. Leukemia.

[13] Muris JJF, Meijer CJLM, Vos W, van Krieken JHJM, Jiwa NM, Ossenkoppele GJ (2006). Immunohistochemical profiling based on Bcl-2, CD10 and MUM1 expression improves risk stratification in patients with primary nodal diffuse large B cell lymphoma. J Pathol.

[14] Natkunam Y, Farinha P, Hsi ED, Hans CP, Tibshirani R, Sehn LH (2008). LMO2 protein expression predicts survival in patients with diffuse large B-cell lymphoma treated with anthracycline-based chemotherapy with and without rituximab. J Clin Oncol.

[15] Coutinho R, Clear AJ, Owen A, Wilson A, Matthews J, Lee A (2013). Poor concordance among nine immunohistochemistry classifiers of cell-of-origin for diffuse large B-cell lymphoma: implications for therapeutic strategies. Clin Cancer Res.

[16] Sujobert P, Salles G, Bachy E (2016). Molecular classification of diffuse large B-cell lymphoma: what is clinically relevant?. Hematol Oncol Clin.

[17] Ott G, Ziepert M, Klapper W, Horn H, Szczepanowski M, Bernd H-W (2010). Immunoblastic morphology but not the immunohistochemical GCB/nonGCB classifier predicts outcome in diffuse large B-cell lymphoma in the RICOVER-60 trial of the DSHNHL. Blood.

[18] Lu T-X, Miao Y, Wu J-Z, Gong Q-X, Liang J-H, Wang Z (2016). The distinct clinical features and prognosis of the CD10^+^MUM1^+^ and CD10^−^ Bcl6^−^ MUM1^−^ diffuse large B-cell lymphoma. Sci Rep.

[19] Huang J-J, Xia Y, Wang Y, Liu P-P, Bi X-W, Sun P (2016). A comparison of R-EPOCH and R-CHOP as a first-line regimen in de novo DLBCL patients with high Ki-67 expression in a single institution. Oncotarget.

[20] Huang D, Quan Y, He M, Zhou B (2009). Comparison of linear discriminant analysis methods for the classification of cancer based on gene expression data. J Exp Clin Cancer Res.

[21] Morais CLM, Lima KMG, Morais CLM, Lima KMG (2018). Principal component analysis with linear and quadratic discriminant analysis for identification of cancer samples based on mass spectrometry. J Braz Chem Soc.

[22] Eberle FC, Rodriguez-Canales J, Wei L, Hanson JC, Killian JK, Sun H-W (2011). Methylation profiling of mediastinal gray zone lymphoma reveals a distinctive signature with elements shared by classical Hodgkin’s lymphoma and primary mediastinal large B-cell lymphoma. Haematologica.

[23] Jais J-P, Molina TJ, Ruminy P, Gentien D, Reyes C, Scott DW (2017). Reliable subtype classification of diffuse large B-cell lymphoma samples from GELA LNH2003 trials using the Lymph2Cx gene expression assay. Haematologica.

[24] Xue X, Zeng N, Gao Z, Du M-Q (2015). Diffuse large B-cell lymphoma: sub-classification by massive parallel quantitative RT-PCR. Lab Invest J Tech Methods Pathol.

[25] Tarca AL, Carey VJ, Chen X, Romero R, Drăghici S (2007). Machine learning and its applications to biology. PLoS Comput Biol.

[26] Whiting P, Martin RM, Ben-Shlomo Y, Gunnell D, Sterne JAC (2013). How to apply the results of a research paper on diagnosis to your patient. JRSM Short Rep.

[27] Frank E, Hall MA, Witten IH (2016). The WEKA Workbench. Online Appendix for “Data mining: practical machine learning tools and techniques.

[28] Pedregosa F, Varoquaux G, Gramfort A, Michel V, Thirion B, Grisel O (2011). Scikit-learn: machine learning in Python. J Mach Learn Res.

[29] Alizadeh AA, Eisen MB, Davis RE, Ma C, Lossos IS, Rosenwald A (2000). Distinct types of diffuse large B-cell lymphoma identified by gene expression profiling. Nature.

[30] Lenz G, Wright G, Dave SS, Xiao W, Powell J, Zhao H (2008). Stromal gene signatures in large-B-cell lymphomas. N Engl J Med.

[31] Rosenwald A, Wright G, Chan WC, Connors JM, Campo E, Fisher RI (2002). The use of molecular profiling to predict survival after chemotherapy for diffuse large-B-cell lymphoma. N Engl J Med.

[32] Batlle-López A, de Villambrosía SG, Francisco M, Malatxeberria S, Sáez A, Montalban C (2016). Stratifying diffuse large B-cell lymphoma patients treated with chemoimmunotherapy: GCB/non-GCB by immunohistochemistry is still a robust and feasible marker. Oncotarget.

[33] Hatzi K, Melnick A (2014). Breaking bad in the germinal center: how deregulation of BCL6 contributes to lymphomagenesis. Trends Mol Med.

[34] Anderson JJ, Fordham S, Overman L, Dignum H, Wood K, Proctor SJ (2009). Immunophenotyping of diffuse large B-cell lymphoma (DLBCL) defines multiple sub-groups of germinal centre-like tumours displaying different survival characteristics. Int J Oncol.

[35] Chang C-C, McClintock S, Cleveland RP, Trzpuc T, Vesole DH, Logan B (2004). Immunohistochemical expression patterns of germinal center and activation B-cell markers correlate with prognosis in diffuse large B-cell lymphoma. Am J Surg Pathol.

[36] Falini B, Fizzotti M, Pucciarini A, Bigerna B, Marafioti T, Gambacorta M (2000). A monoclonal antibody (MUM1p) detects expression of the MUM1/IRF4 protein in a subset of germinal center B cells, plasma cells, and activated T cells. Blood.

[37] Schmitz R, Wright GW, Huang DW, Johnson CA, Phelan JD, Wang JQ (2018). Genetics and pathogenesis of diffuse large B-cell lymphoma. N Engl J Med.

[38] Chapuy B, Stewart C, Dunford AJ, Kim J, Kamburov A, Redd RA (2018). Molecular subtypes of diffuse large B cell lymphoma are associated with distinct pathogenic mechanisms and outcomes. Nat Med.

[39] Gutiérrez-García G, Cardesa-Salzmann T, Climent F, González-Barca E, Mercadal S, Mate JL (2011). Gene-expression profiling and not immunophenotypic algorithms predicts prognosis in patients with diffuse large B-cell lymphoma treated with immunochemotherapy. Blood.

[40] Liu Y, Barta SK (2019). Diffuse large B-cell lymphoma: 2019 update on diagnosis, risk stratification, and treatment. Am J Hematol.

[41] Tilly H, Gomes da Silva M, Vitolo U, Jack A, Meignan M, Lopez-Guillermo A (2015). Diffuse large B-cell lymphoma (DLBCL): ESMO Clinical Practice Guidelines for diagnosis, treatment and follow-up. Ann Oncol.

[42] Molina TJ, Canioni D, Copie-Bergman C, Recher C, Brière J, Haioun C (2014). Young patients with non-germinal center B-cell-like diffuse large B-cell lymphoma benefit from intensified chemotherapy with ACVBP plus rituximab compared with CHOP plus rituximab: analysis of data from the Groupe d’Etudes des Lymphomes de l’Adulte/lymphoma study association phase III trial LNH 03-2B. J Clin Oncol.

[43] Offner F, Samoilova O, Osmanov E, Eom H-S, Topp MS, Raposo J (2015). Frontline rituximab, cyclophosphamide, doxorubicin, and prednisone with bortezomib (VR-CAP) or vincristine (R-CHOP) for non-GCB DLBCL. Blood.

[44] Leonard JP, Kolibaba KS, Reeves JA, Tulpule A, Flinn IW, Kolevska T (2017). Randomized phase II study of R-CHOP with or without bortezomib in previously untreated patients with non-germinal center B-cell-like diffuse large B-cell lymphoma. J Clin Oncol.

