# Supplementary material for: Discriminant analysis and machine learning approach for evaluating and improving the performance of immunohistochemical algorithms for COO classification of DLBCL
Source: J Transl Med. 2019 Jun 11;17:198. doi: 10.1186/s12967-019-1951-y (PMC6560900; doi:10.1186/s12967-019-1951-y)
Supplement: Supplementary file 1 — Additional file 1. Additional Figures S1, S2 and Tables S1–S6. [file 12967_2019_1951_MOESM1_ESM.docx]

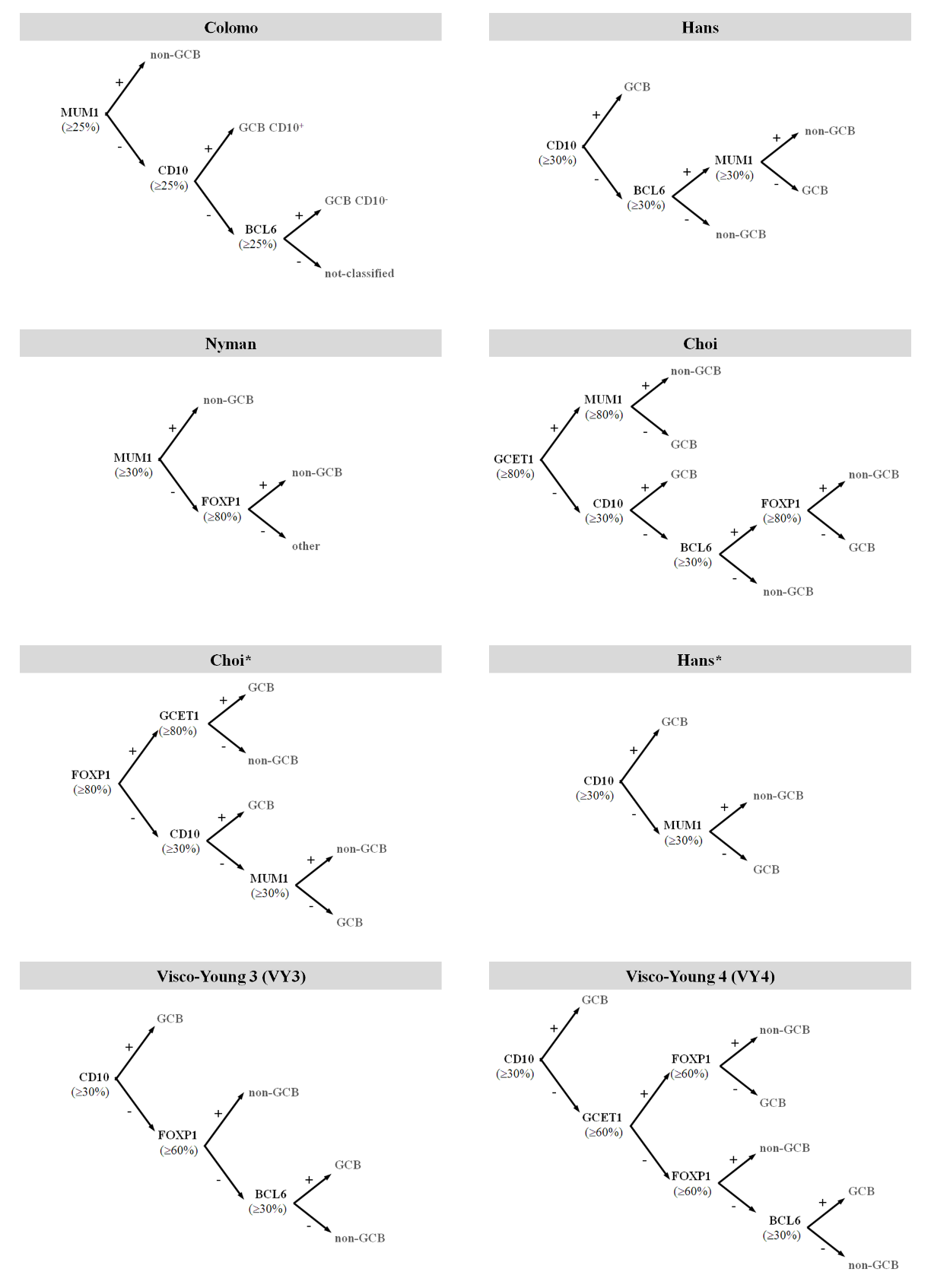


**Figure S1. Immunohistochemical (IHC)-based decision tree algorithms used to classify DLBCL into molecular subgroups**. Eight IHC-decision tree algorithms were proven to identify the cell-of-origin in DLBCL cases. GCB, germinal center B-cell type. Branches are result of a specific threshold.

**Table S1. Feature combination for the IHC-decision trees and machine learning algorithms.** Both IHC-decision trees and machine learning algorithms used the same immunohistochemistry staining data (antibody combination) as the input, e.g. VY3 used CD10, BCL6, and FOXP1 data, that is (1,2,3), as well as the ANN (1,2,3).

|  | **Antibody** | | | | |  |
| --- | --- | --- | --- | --- | --- | --- |
|  | **1** | **2** | **3** | **4** | **5** |  |
| **Algorithm** | **CD10** | **BCL6** | **FOXP1** | **GCET1** | **MUM1** | **Combination** |
| Nyman |  |  | ⚫ |  | ⚫ | 3,5 |
| Colomo | ⚫ | ⚫ |  |  | ⚫ | 1,2,5 |
| Hans | ⚫ | ⚫ |  |  | ⚫ | 1,2,5 |
| Hans* | ⚫ |  |  |  | ⚫ | 1,5 |
| Choi | ⚫ | ⚫ | ⚫ | ⚫ | ⚫ | 1,2,3,4,5 |
| Choi* | ⚫ |  | ⚫ | ⚫ | ⚫ | 1,3,4,5 |
| VY3 | ⚫ | ⚫ | ⚫ |  |  | 1,2,3 |
| VY4 | ⚫ | ⚫ | ⚫ | ⚫ |  | 1,2,3,4 |

**Table S2. Comparison of antibodies and dilutions used for immunohistochemical analysis.**

|  | Current study | | | Visco *et.al.* | | |
| --- | --- | --- | --- | --- | --- | --- |
| Antibody | **Supplier** | **Clone** | **Dilution** | **Supplier** | **Clone** | **Dilution** |
| CD10 | Santa Cruz Biotechnology | 56C6 | 1:10 | Novocastra | 56C6 | 1:100 |
| BCL6 | Santa Cruz Biotechnology | 0.N.26 | 1:5 | Novocastra | LN22 | 1:100 |
| FOXP1 | abcam | JC12 | 1:300 | abcam | JC12 | 1:16,000 |
| GCTE1 | abcam | RAM341 | 1:100 | abcam | RAM341 | 1:4 |
| MUM1 | Santa Cruz Biotechnology | monoclonal | 1:50 | Dako | MUMp1 | 1:20 |

**Suplement Table S3. Baseline characteristics of 49 DLBCL Mexican patients in the clinical sample set.**

| **Characteristic** | **n (%)** |
| --- | --- |
| Gender (M:F ratio) | 26:23 (1.1:1) |
| Median age | 60.3 (21 – 100) |
| Age > 60 y (n[%]) | 27 (55.1%) |
| ECOG performance status (n[%]) |  |
| 0-2 | 45 (91.8%) |
| ≥ 3 | 3 (6.1%) |
| not evaluated | 1 (2.0%) |
| Ann Arbor stage (n[%]) |  |
| I-II | 25 (51.0%) |
| III-IV | 23 (46.9%) |
| not evaluated | 1 (2.0%) |
| Extranodal involvement ≥ 2 (n[%]) | 18 (36.7%) |
| B symptoms (n[%]) | 34 (69.4%) |
| Serum LDH elevation (n[%]) | 27 (55.1%) |
| not evaluated | 7 (14.3%) |
| IPI (n[%]) |  |
| Low risk (L) | 12 (24.5%) |
| Low-intermediate risk (Li) | 15 (30.6%) |
| High-intermediate risk (Hi) | 10 (20.4%) |
| High risk (H) | 5 (10.2%) |
| unknown | 7 (14.3%) |
| NCCN-IPI (n[%]) |  |
| Low risk (L) | 5 (10.2%) |
| Low-intermediate risk (Li) | 21 (42.9%) |
| High-intermediate risk (Hi) | 11 (22.4%) |
| High risk (H) | 5 (10.2%) |
| unknown | 7 (14.3%) |
| Treatment regimen (n[%]) |  |
| anthracycline-based | 18 (36.7%) |
| R- anthracycline-based | 21 (42.9%) |
| other | 10 (20.4%) |
| Treatment response (n[%]) |  |
| CR | 24 (49.0%) |
| PR | 10 (20.4%) |
| PD | 4 (8.2%) |
| unknown | 11 (22.4%) |
| death | 13 (26.5%) |
| Relapse after CR (n[%]) | 6 (12.2%) |
| Median follow-up (range) (months) | 30.3 (0.1– 167.8) |

ECOG, Eastern Cooperative Oncology Group. LDH, lactate dehydrogenase. IPI, International Prognostic Index. R-IPI, revised IPI NCCN-IPI, National Comprehensive Cancer Network IPI. R-, treatment with rituximab and chemotherapy. CR, complete remission. PR, partial response. PD, progressive disease**.**

**Table S4. Performance metrics of classification of IHC-decision tree algorithms and LDA.** The upper section corresponds to the performance of the IHC-decision tree algorithms. The lower section corresponds to equivalent combinations of antibodies, but with LDA classification, this includes the rest of combinations not reported by IHC-decision tree algorithms. Numeric Tags 1= CD10, 2 = BCL6, 3 = FOXP1, 4 = GCTE1, and 5 = MUM1.

|  |  | **Algorithm** | **Antibody combination** | **Acc** | **Sens** | **Spec** | **PPV** | **NPV** | **LR+** | **LR-** | |
| --- | --- | --- | --- | --- | --- | --- | --- | --- | --- | --- | --- |
| IHC-decision tree |  | Nyman | 3,5 | 0.72 | 0.52 | 0.91 | 0.84 | 0.67 | 5.56 | 0.53 | |
|  |  | Colomo | 1,2,5 | 0.78 | 0.71 | 0.84 | 0.81 | 0.75 | 4.56 | 0.34 | |
|  |  | **Hans** | **1,2,5** | **0.85** | **0.91** | **0.78** | **0.80** | **0.91** | **4.21** | **0.11** | |
|  |  | Hans* | 1,5 | 0.82 | 0.94 | 0.70 | 0.75 | 0.92 | 3.14 | 0.09 | |
|  |  | Choi | 1,2,3,4,5 | 0.88 | 0.94 | 0.84 | 0.84 | 0.93 | 5.70 | 0.08 | |
|  |  | Choi* | 1,3,4,5 | 0.79 | 0.74 | 0.83 | 0.80 | 0.77 | 4.30 | 0.31 | |
|  |  | VY3 | 1,2,3 | 0.88 | 0.92 | 0.84 | 0.85 | 0.92 | 5.92 | 0.09 | |
|  |  | VY4 | 1,2,3,4 | 0.88 | 0.93 | 0.84 | 0.85 | 0.92 | 5.80 | 0.09 | |
|  |  |  |  |  |  |  |  |  |  |  | |
| Linear Discriminant Analysis |  |  | 1,2 | 0.82 | 0.72 | 0.91 | 0.88 | 0.77 | 7.97 | 0.31 | |
|  |  |  | 1,3 | 0.84 | 0.79 | 0.88 | 0.86 | 0.82 | 6.44 | 0.24 | |
|  |  |  | 1,4 | 0.83 | 0.74 | 0.91 | 0.89 | 0.79 | 8.65 | 0.28 | |
|  |  | Hans* | **1,5** | 0.84 | 0.77 | 0.91 | 0.89 | 0.81 | 8.59 | 0.25 | |
|  |  |  | 2,3 | 0.76 | 0.66 | 0.84 | 0.80 | 0.73 | 4.25 | 0.40 | |
|  |  |  | 2,4 | 0.73 | 0.62 | 0.84 | 0.79 | 0.70 | 3.97 | 0.45 | |
|  |  |  | 2,5 | 0.79 | 0.83 | 0.75 | 0.76 | 0.83 | 3.38 | 0.22 | |
|  |  |  | 3,4 | 0.76 | 0.75 | 0.77 | 0.75 | 0.76 | 3.21 | 0.33 | |
|  |  | Nyman | **3,5** | 0.77 | 0.81 | 0.74 | 0.75 | 0.81 | 3.10 | 0.25 | |
|  |  |  | 4,5 | 0.80 | 0.84 | 0.76 | 0.77 | 0.83 | 3.46 | 0.22 | |
|  |  | VY3 | **1,2,3** | 0.89 | 0.87 | 0.91 | 0.90 | 0.88 | 9.19 | 0.15 | |
|  |  |  | 1,2,4 | 0.83 | 0.74 | 0.91 | 0.88 | 0.79 | 7.85 | 0.29 | |
|  |  | Hans/Colomo | **1,2,5** | 0.87 | 0.86 | 0.88 | 0.87 | 0.87 | 7.25 | 0.16 | |
|  |  |  | 1,3,4 | 0.86 | 0.80 | 0.91 | 0.90 | 0.83 | 9.31 | 0.22 | |
|  |  |  | 1,3,5 | 0.86 | 0.84 | 0.87 | 0.86 | 0.85 | 6.61 | 0.18 | |
|  |  |  | **1,4,5** | 0.87 | 0.81 | 0.92 | 0.90 | 0.84 | 9.93 | 0.20 | |
|  |  |  | 2,3,4 | 0.81 | 0.75 | 0.86 | 0.84 | 0.79 | 5.57 | 0.29 | |
|  |  |  | 2,3,5 | 0.84 | 0.85 | 0.83 | 0.83 | 0.85 | 5.05 | 0.18 | |
|  |  |  | 2,4,5 | 0.83 | 0.81 | 0.84 | 0.83 | 0.82 | 5.20 | 0.23 | |
|  |  |  | 3,4,5 | 0.82 | 0.79 | 0.84 | 0.83 | 0.81 | 5.06 | 0.25 | |
|  |  | VY4 | **1,2,3,4** | 0.87 | 0.84 | 0.90 | 0.89 | 0.86 | 8.24 | 0.17 | |
|  |  |  | 1,2,3,5 | 0.88 | 0.85 | 0.90 | 0.89 | 0.87 | 8.32 | 0.16 | |
|  |  |  | 1,2,4,5 | 0.86 | 0.82 | 0.91 | 0.89 | 0.84 | 8.68 | 0.20 | |
|  |  | Choi* | **1,3,4,5** | 0.88 | 0.86 | 0.91 | 0.90 | 0.87 | 9.09 | 0.16 | |
|  |  |  | 2,3,4,5 | 0.85 | 0.84 | 0.86 | 0.86 | 0.85 | 6.24 | 0.18 | |
|  |  | Choi | **1,2,3,4,5** | 0.89 | 0.87 | 0.91 | 0.90 | 0.88 | 9.23 | 0.14 | |
|  |  |  |  |  |  |  |  |  |  |  | |
|  |  | Acc accuracy, Sens sensitivity, Spec specificity, PPV positive predictive value, NPV negative predictive values, LR+ likelihood ratio for positive test results, LR- likelihood ratio for negative test result. | | | | | | | | |  |

**Table S5. Coefficients of Linear Discriminant Functions (LDF) derived from LDA for all possible combination of antibodies.** Columns (Antibody) show the coefficient associated with each antibody for GCB classification (Sensibility performance) and non-GCB classification (Specificity performance). The first column remarks the combinations of antibodies as were used by IHC-decision trees. Numeric Tags 1= CD10, 2 = BCL6, 3 = FOXP1, 4 = GCTE1, and 5 = MUM1.

|  |  |  |  |  |  | **Antibody** | | | | |
| --- | --- | --- | --- | --- | --- | --- | --- | --- | --- | --- |
|  |  |  |  |  |  | **1** | **2** | **3** | **4** | **5** |
| **Antibody combination** | | **Sens** | **Spec** | **COO** | **Constant** | **CD10** | **BCL6** | **FOXP1** | **GCET1** | **MUM1** |
|  | 1,2 | 0.72 | 0.91 | GCB | -4.17 | 4.93 | 7.19 |  |  |  |
|  |  |  |  | non-GCB | -1.53 | -0.24 | 6.05 |  |  |  |
|  | 1,3 | 0.79 | 0.88 | GCB | -2.33 | 6.11 |  | 1.95 |  |  |
|  |  |  |  | non-GCB | -2.1 | 0.09 |  | 5.47 |  |  |
|  | 1,4 | 0.74 | 0.91 | GCB | -2.9 | 5.93 |  |  | 3.87 |  |
|  |  |  |  | non-GCB | -0.14 | 0.87 |  |  | 1.27 |  |
| as in Hans* | 1,5 | 0.77 | 0.91 | GCB | -2.29 | 6.53 |  |  |  | 2.11 |
|  |  |  |  | non-GCB | -2.12 | 1.28 |  |  |  | 6.45 |
|  | 2,3 | 0.66 | 0.84 | GCB | -3.07 |  | 8.06 | 1.02 |  |  |
|  |  |  |  | non-GCB | -2.87 |  | 4.42 | 4.55 |  |  |
|  | 2,4 | 0.62 | 0.84 | GCB | -3.7 |  | 7.82 |  | 3.58 |  |
|  |  |  |  | non-GCB | -1.55 |  | 5.89 |  | 0.62 |  |
|  | 2,5 | 0.83 | 0.75 | GCB | -3.03 |  | 8.3 |  |  | 0.67 |
|  |  |  |  | non-GCB | -3.081 |  | 5.04 |  |  | 5.68 |
|  | 3,4 | 0.75 | 0.77 | GCB | -1.74 |  |  | 2.87 | 4.71 |  |
|  |  |  |  | non-GCB | -2.25 |  |  | 5.53 | 1.61 |  |
| as in Nyman | 3,5 | 0.81 | 0.74 | GCB | -0.57 |  |  | 2.47 |  | 1.11 |
|  |  |  |  | non-GCB | -3.29 |  |  | 4.38 |  | 5.06 |
|  | 4,5 | 0.84 | 0.76 | GCB | -1.37 |  |  |  | 4.64 | 2.02 |
|  |  |  |  | non-GCB | -2.17 |  |  |  | 1.57 | 6.45 |
| as in VY3 | 1,2,3 | 0.87 | 0.91 | GCB | -4.18 | 4.86 | 6.99 | 0.64 |  |  |
|  |  |  |  | non-GCB | -2.9 | -0.74 | 4.58 | 4.61 |  |  |
|  | 1,2,4 | 0.74 | 0.91 | GCB | -4.7 | 4.6 | 6.75 |  | 3.17 |  |
|  |  |  |  | non-GCB | -1.55 | -0.31 | 5.96 |  | 0.65 |  |
| as in Hans | 1,2,5 | 0.86 | 0.88 | GCB | -4.21 | 5.01 | 7 |  |  | 1.05 |
| and Colomo |  |  |  | non-GCB | -3.09 | 0.2 | 4.99 |  |  | 5.69 |
|  | 1,3,4 | 0.8 | 0.91 | GCB | -3.21 | 5.53 |  | 2.15 | 4 |  |
|  |  |  |  | non-GCB | -2.25 | -0.15 |  | 5.55 | 1.63 |  |
|  | 1,3,5 | 0.84 | 0.87 | GCB | -2.46 | 6.24 |  | 1.58 |  | 1.61 |
|  |  |  |  | non-GCB | -3.3 | 0.48 |  | 4.31 |  | 5.1 |
|  | 1,4,5 | 0.81 | 0.92 | GCB | -3.14 | 6.01 |  |  | 3.93 | 2.22 |
|  |  |  |  | non-GCB | -2.23 | 1.09 |  |  | 1.44 | 6.49 |

**Table S5. Coefficients of Linear Discriminant Functions (LDF) derived from LDA for all possible combination of antibodies.** (cont…).

|  |  |  |  |  |  | **Antibody** | | | | |
| --- | --- | --- | --- | --- | --- | --- | --- | --- | --- | --- |
|  |  |  |  |  |  | **1** | **2** | **3** | **4** | **5** |
| **Antibody combination** | | **Sens** | **Spec** | **COO** | **Constant** | **CD10** | **BCL6** | **FOXP1** | **GCET1** | **MUM1** |
|  | 2,3,4 | 0.75 | 0.86 | GCB | -3.81 |  | 7.35 | 1.3 | 3.7 |  |
|  |  |  |  | non-GCB | -2.93 |  | 4.22 | 4.63 | 1.03 |  |
|  | 2,3,5 | 0.85 | 0.83 | GCB | -3.08 |  | 8.02 | 0.94 |  | 0.42 |
|  |  |  |  | non-GCB | -3.9 |  | 3.95 | 3.63 |  | 4.72 |
|  | 2,4,5 | 0.81 | 0.84 | GCB | -3.75 |  | 7.66 |  | 3.63 | 0.88 |
|  |  |  |  | non-GCB | -3.13 |  | 4.87 |  | 0.92 | 5.72 |
|  | 3,4,5 | 0.79 | 0.84 | GCB | -1.81 |  |  | 2.6 | 4.73 | 1.23 |
|  |  |  |  | non-GCB | -3.45 |  |  | 4.43 | 1.71 | 5.1 |
| as in VY4 | 1,2,3,4 | 0.84 | 0.9 | GCB | -4.75 | 4.49 | 6.44 | 0.91 | 3.26 |  |
|  |  |  |  | non-GCB | -2.97 | -0.86 | 4.4 | 4.7 | 1.12 |  |
|  | 1,2,3,5 | 0.85 | 0.9 | GCB | -4.22 | 4.95 | 6.87 | 0.46 |  | 0.92 |
|  |  |  |  | non-GCB | -3.9 | -0.27 | 4.01 | 3.65 |  | 4.69 |
|  | 1,2,4,5 | 0.82 | 0.91 | GCB | -4.77 | 4.69 | 6.51 |  | 3.22 | 1.21 |
|  |  |  |  | non-GCB | -3.13 | 0.1 | 4.85 |  | 0.91 | 5.73 |
| as in Choi* | 1,3,4,5 | 0.86 | 0.91 | GCB | -3.34 | 5.67 |  | 1.78 | 4.03 | 1.67 |
|  |  |  |  | non-GCB | -3.46 | 0.24 |  | 4.39 | 1.69 | 5.12 |
|  | 2,3,4,5 | 0.84 | 0.86 | GCB | -3.83 |  | 7.29 | 1.18 | 3.72 | 0.57 |
|  |  |  |  | non-GCB | -3.98 |  | 3.71 | 3.71 | 1.2 | 4.77 |
| as in Choi | 1,2,3,4,5 | 0.87 | 0.91 | GCB | -4.8 | 4.59 | 6.31 | 0.71 | 3.28 | 1.02 |
|  |  |  |  | non-GCB | -3.98 | -0.41 | 3.8 | 3.75 | 1.24 | 4.73 |


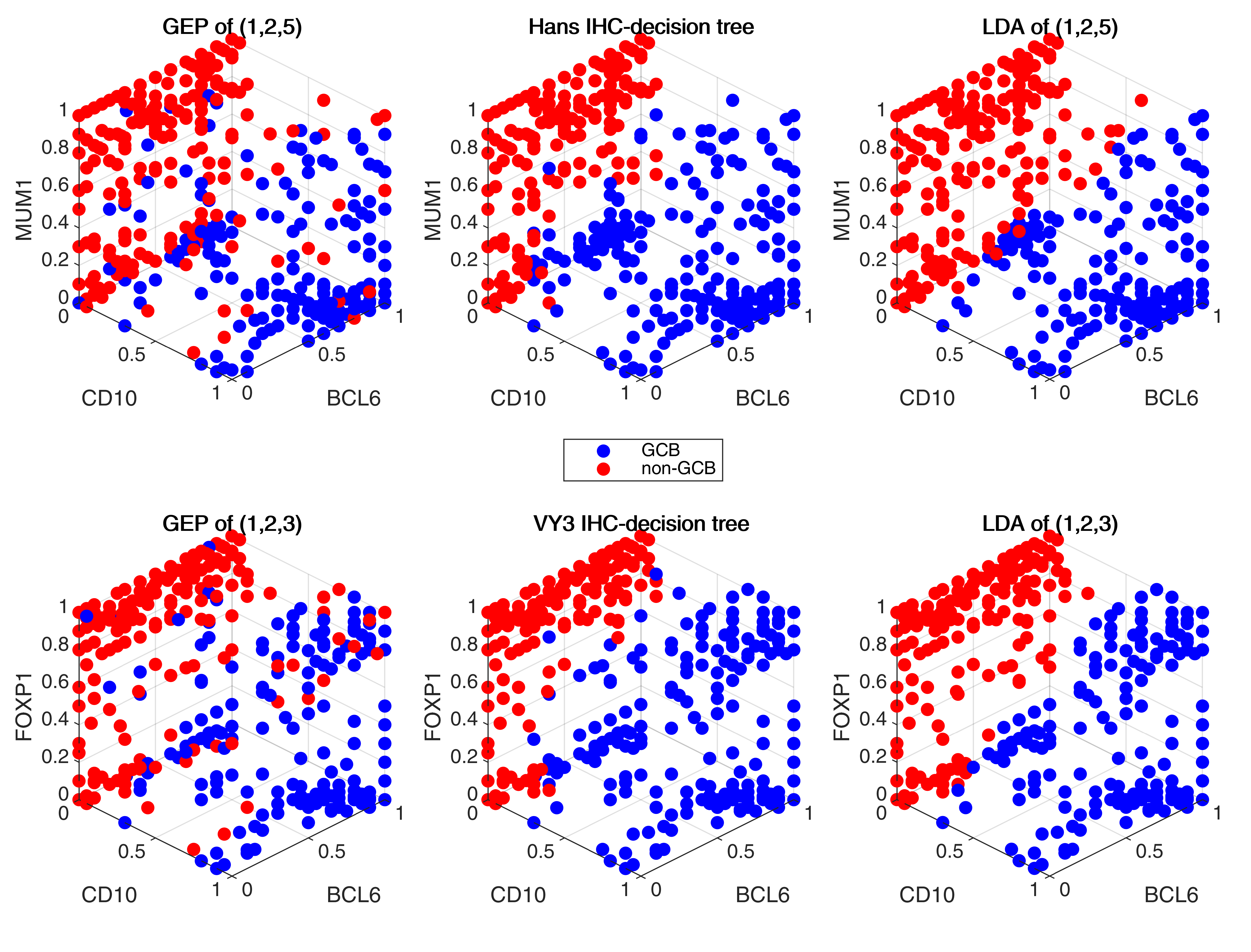


**Figure S2. DLBCL COO group separation using IHC-decision tree and LDA.** Using Hans and VY3 antibody combination as example, graphs show the immunohistochemistry staining data and the performance of GCB (blue) and non-GCB (red) cases, classified with GEP, IHC-decision trees, and LDA analysis. The separation of COO groups increases using LDA in comparison to IHC-decision trees. In this case, LDA of (1,2,3) is better separating COO groups compared with LDA of (1,2,5). GEP shows no separation since it is the true class and relies in the genome-wide expression. Numeric Tags 1= CD10, 2 = BCL6, 3 = FOXP1, 4 = GCTE1, and 5 = MUM1.

**Table S6. Performance metrics of classification with IHC-decision tree algorithms; and machine learning algorithms.** Metrics correspondent to eight IHC-decision tree and 35 machine learning algorithms are shown, cases of the VY subset were classified. Numeric Tags 1= CD10, 2 = BCL6, 3 = FOXP1, 4 = GCTE1, and 5 = MUM1.

|  |  | **Algorithm** | **Antibody combination** | **Acc** | **Sens** | **Spec** | **PPV** | **NPV** | **LR+** | **LR-** | |
| --- | --- | --- | --- | --- | --- | --- | --- | --- | --- | --- | --- |
| IHC-decision tree |  | Nyman | 3,5 | 0.79 | 0.65 | 0.95 | 0.93 | 0.71 | 12.47 | 0.37 | |
|  |  | Colomo | 1,2,5 | 0.84 | 0.77 | 0.91 | 0.91 | 0.79 | 8.98 | 0.25 | |
|  |  | **Hans** | **1,2,5** | **0.89** | **0.95** | **0.83** | **0.86** | **0.94** | **5.52** | **0.06** | |
|  |  | Hans* | 1,5 | 0.86 | 0.95 | 0.76 | 0.81 | 0.94 | 3.94 | 0.06 | |
|  |  | Choi | 1,2,3,4,5 | 0.93 | 1.00 | 0.84 | 0.87 | 1.00 | 6.44 | 0.00 | |
|  |  | Choi* | 1,3,4,5 | 0.83 | 0.79 | 0.86 | 0.86 | 0.79 | 5.73 | 0.24 | |
|  |  | VY3 | 1,2,3 | 0.90 | 0.97 | 0.83 | 0.86 | 0.96 | 5.61 | 0.04 | |
|  |  | VY4 | 1,2,3,4 | 0.90 | 0.97 | 0.83 | 0.86 | 0.96 | 5.61 | 0.04 | |
|  |  |  |  |  |  |  |  |  |  |  | |
| Machine learning algorithms |  | B | 3,5 | 0.84 | 0.87 | 0.81 | 0.83 | 0.85 | 4.59 | 0.16 | |
|  |  |  | 1,2,5 | 0.90 | 0.89 | 0.91 | 0.92 | 0.88 | 10.29 | 0.12 | |
|  |  |  | 1,5 | 0.83 | 0.74 | 0.93 | 0.92 | 0.77 | 10.76 | 0.28 | |
|  |  |  | 1,2,3,4,5 | 0.93 | 0.95 | 0.91 | 0.92 | 0.95 | 11.04 | 0.05 | |
|  |  |  | 1,3,4,5 | 0.93 | 0.94 | 0.93 | 0.94 | 0.93 | 13.56 | 0.07 | |
|  |  |  | 1,2,3 | 0.89 | 0.89 | 0.9 | 0.9 | 0.88 | 8.58 | 0.13 | |
|  |  |  | 1,2,3,4 | 0.88 | 0.87 | 0.88 | 0.89 | 0.86 | 7.22 | 0.15 | |
|  |  | BN | 3,5 | 0.84 | 0.77 | 0.91 | 0.91 | 0.79 | 8.98 | 0.25 | |
|  |  |  | 1,2,5 | 0.89 | 0.87 | 0.91 | 0.92 | 0.87 | 10.1 | 0.14 | |
|  |  |  | 1,5 | 0.88 | 0.9 | 0.86 | 0.88 | 0.89 | 6.55 | 0.11 | |
|  |  |  | 1,2,3,4,5 | 0.93 | 0.94 | 0.91 | 0.92 | 0.93 | 10.85 | 0.07 | |
|  |  |  | 1,3,4,5 | 0.91 | 0.92 | 0.90 | 0.9 | 0.91 | 8.89 | 0.09 | |
|  |  |  | 1,2,3 | 0.89 | 0.90 | 0.88 | 0.89 | 0.89 | 7.48 | 0.11 | |
|  |  |  | 1,2,3,4 | 0.86 | 0.87 | 0.84 | 0.86 | 0.86 | 5.61 | 0.15 | |
|  |  | BS | 3,5 | 0.85 | 0.89 | 0.81 | 0.83 | 0.87 | 4.68 | 0.14 | |
|  |  |  | 1,2,5 | 0.89 | 0.89 | 0.90 | 0.90 | 0.88 | 8.58 | 0.13 | |
|  |  |  | 1,5 | 0.83 | 0.74 | 0.93 | 0.92 | 0.77 | 10.76 | 0.28 | |
|  |  |  | **1,2,3,4,5** | **0.94** | **0.95** | **0.93** | **0.94** | **0.95** | **13.8** | **0.05** | |
|  |  | **PV** | **1,3,4,5** | **0.94** | **0.95** | **0.93** | **0.94** | **0.95** | **13.8** | **0.05** | |
|  |  |  | 1,2,3 | 0.90 | 0.90 | 0.90 | 0.90 | 0.90 | 8.73 | 0.11 | |
|  |  |  | 1,2,3,4 | 0.88 | 0.87 | 0.88 | 0.89 | 0.86 | 7.22 | 0.15 | |
|  |  | ANN | 3,5 | 0.83 | 0.85 | 0.81 | 0.83 | 0.84 | 4.51 | 0.18 | |
|  |  |  | 1,2,5 | 0.91 | 0.94 | 0.88 | 0.89 | 0.93 | 7.75 | 0.07 | |
|  |  |  | 1,5 | 0.89 | 0.84 | 0.95 | 0.95 | 0.85 | 16.22 | 0.17 | |
|  |  |  | **1,2,3,4,5** | **0.94** | **0.95** | **0.93** | **0.94** | **0.95** | **13.8** | **0.05** | |
|  |  |  | 1,3,4,5 | 0.93 | 0.92 | 0.93 | 0.93 | 0.92 | 13.33 | 0.09 | |
|  |  |  | 1,2,3 | 0.92 | 0.94 | 0.90 | 0.91 | 0.93 | 9.04 | 0.07 | |
|  |  |  | 1,2,3,4 | 0.90 | 0.92 | 0.88 | 0.89 | 0.91 | 7.62 | 0.09 | |
|  |  | SVM | 3,5 | 0.83 | 0.90 | 0.76 | 0.80 | 0.88 | 3.74 | 0.13 | |
|  |  |  | 1,2,5 | 0.90 | 0.89 | 0.91 | 0.92 | 0.88 | 10.29 | 0.12 | |
|  |  |  | 1,5 | 0.89 | 0.90 | 0.88 | 0.89 | 0.89 | 7.48 | 0.11 | |
|  |  |  | **1,2,3,4,5** | **0.94** | **0.97** | **0.91** | **0.92** | **0.96** | **11.23** | **0.04** | |
|  |  |  | 1,3,4,5 | 0.93 | 0.95 | 0.90 | 0.91 | 0.95 | 9.20 | 0.05 | |
|  |  |  | 1,2,3 | 0.89 | 0.92 | 0.86 | 0.88 | 0.91 | 6.67 | 0.09 | |
|  |  |  | **1,2,3,4** | **0.94** | **0.97** | **0.91** | **0.92** | **0.96** | **11.23** | **0.04** | |
|  |  |  |  |  |  |  |  |  |  |  | |
|  |  | Acc accuracy, Sens sensitivity, Spec specificity, PPV positive predictive value, NPV negative predictive values, LR+ likelihood ratio for positive test results, LR- likelihood ratio for negative test result. | | | | | | | | |  |
